# RNA N^6^-adenosine methylation (m^6^A) steers epitranscriptomic control of herpesvirus replication

**Published:** 2017-10-17

**Authors:** Fengchun Ye

**Affiliations:** Department of Microbiology and Molecular Biology, School of Medicine, Case Western Reserve University, 10900 Euclid avenue, Cleveland, 44106 Ohio, USA

**Keywords:** RNA m^6^A modification, RTA pre-mRNA splicing, KSHV lytic replication, epitranscriptomics

## Abstract

Latency is a hallmark of all herpesviruses, during which the viral genomes are silenced through DNA methylation and suppressive histone modifications. When latent herpesviruses reactivate to undergo productive lytic replication, the suppressive epigenetic marks are replaced with active ones to allow for transcription of viral genes. Interestingly, by using Kaposi’s sarcoma-associated herpesvirus (KSHV) as a model, we recently demonstrated that the newly transcribed viral RNAs are also subjected to post-transcriptional N^6^-adenosine methylation (m^6^A). Blockade of this post-transcriptional event abolishes viral protein expression and halts virion production. We found that m^6^A modification controls RNA splicing, stability, and protein translation to regulate viral lytic gene expression and replication. Thus, our finding for the first time reveals a critical role of this epitranscriptomic mechanism in the control of herpesviral replication, which shall shed lights on development of novel strategies for the control of herpesviral infection.

Kaposi’s sarcoma-associated herpesvirus (KSHV) is an oncogenic virus associated with multiple malignancies including Kaposi’s sarcoma (KS), primary effusion lymphoma (PEL), and multicentric Castleman’s disease (MCD)^[1–3]^. Like all herpesviruses, KSHV enters a latent phase shortly after primary infection. Under immune suppressive conditions, the latent virus reactivates to undergo lytic replication to produce new viruses. Productive lytic replication not only causes *de novo* infection but also plays an essential role in the development of KS and MCD^[4, 5]^. Previous studies demonstrate that the switch from latency to lytic replication is primarily controlled at the viral chromatin level through epigenetic mechanisms^[6, 7]^. Indeed, the majority of KSHV genome is silenced during latency through DNA methylation, repressive histone modifications, and other negative gene expression regulatory mechanisms^[7–11]^. When the latent virus reactivates, prompt epigenetic changes occur, leading to transactivation of the viral genome. However, our recent study discovered that KSHV reactivation stalls if the newly transcribed viral RNAs fail to undergo post-transcriptional N^6^-adenosine methylation (m^6^A)^[12]^. Our finding highlights a pivotal role of this epitranscriptomic mechanism in the control of KSHV lytic replication.

RNA N^6^-adenosine methylation (m^6^A) is one of the most abundant types of RNA modifications found in over 25% of RNA species in mammalian cells^[13–15]^. A complex of three methyltransferases: methyltransferase like 3 (METTL3), methyltransferase like 14 (METTL14), and Wilms tumor 1 associated protein (WTAP) acts as m^6^A writers and catalyze RNA m^6^A at specific sites with the consensus sequence (G/AGAC)^[16–18]^. Two demethylases, fat mass and obesity associated protein (FTO), and AlkB Homolog 5 (ALKBH5), act as m^6^A erasers and reverse this process^[19–21]^. Most m^6^A sites are located near the transcription start sites, exonic regions flanking splicing sites, stop codons, and the 3’untranslated region (3’UTR)^[14, 22–24]^. The biological functions of m^6^A are mediated by m^6^A readers. In the nucleus, for example, heterogeneous nuclear ribonucleoproteins hn-RNP-C and hn-RNP-A2/B1 selectively bind RNA at m^6^A sites to regulate pre-mRNA processing and alternative splicing^[22, 24–27]^. In addition, the YTH domain containing 1 protein (YTHDC1) binds pre-mRNA at m^6^A sites and preferentially recruits the serine/arginine-rich splicing factor 3 (SRSF3) over SRSF10 for exon inclusion splicing^[28–31]^. In the cytoplasm, three members of the YTH domain-containing family proteins, YTHDF1, YTHDF2, and YTHDF3, preferentially bind m^6^A-containing mRNAs to regulate RNA stability, protein translation, and RNA decay^[32–35]^. In addition, the eIF3, a component of 43S translation pre-initiation complex^[36]^, directly binds m^6^A sites in the 5’untranslated region (5’UTR) of mRNAs to enhance protein translation^[37]^. Therefore, m^6^A represents a very important cellular mechanism for the control of gene expression at the post-transcriptional level. Interestingly, massive increases in m^6^A modification occur in the RNAs of human immunodeficiency virus-1 (HIV-1)^[38, 39]^. Blockade of m^6^A effectively abolishes HIV-1 protein expression and virion production, suggesting that this epitranscriptomic mechanism also controls viral gene expression.

Similar to HIV-1, most KSHV transcripts undergo m^6^A modification, and the level of m^6^A-modified mRNA of a given viral transcript increases in parallel with that of total mRNA when latently infected cells are induced by phorbol ester (TPA) or other lytic replication stimuli. Expressional knocking down of the m^6^A writer METTL3 substantially reduces TPA induction of KSHV lytic genes, and blockade of m^6^A reaction literally abolishes expression of all lytic genes examined and halts virion production. In contrast, expressional knocking down or activity inhibition of the m^6^A eraser FTO has the opposite effects.

To understand how RNA methylation controls KSHV replication, we examined the effect of m^6^A on expression of viral regulator of transcription activation (RTA), which, encoded by open reading frame 50 (ORF50), is a key mediator of the switch from latency to lytic gene expression^[40]^. Due to differential splicing, the ORF50 (RTA) and ORFK8 loci produce at least three different groups of transcripts, including ORF50 /ORFK8/ORFK8.1 tricistronic mRNAs, ORFK8/ORFK8.1 bicistronic mRNAs, and monocistronic ORFK8.1 mRNAs^[41]^. RTA, which is expressed from the tricistronic mRNAs, consists of two exons and one intron (Fig. 1). Interestingly, blockade of m^6^A substantially reduces the level of TPA-induced RTA mRNA but has much less an effect on the level of RTA pre-mRNA, suggesting that m^6^A controls RTA pre-mRNA splicing. Indeed, multiple m^6^A sites are identified in RTA pre-mRNA. Data from genetic mutation assays demonstrate that the m^6^A sites in the intron near the two splicing sites are critical for RTA expression, and one m^6^A site in Exon2 near the splicing site also plays an important role in RTA pre-mRNA splicing. Data from RNA immuno-precipitation (RIP) assays confirm that these sites are indeed m^6^A modified. In addition, both SRSF3 and SRSF10 are present at the m^6^A sites in the intron near the two splicing sites, and the levels of m^6^A and these splicing factors increase significantly upon TPA treatment. Mutation of these m^6^A sites abolishes the RNA-protein interactions and RTA protein expression, thus suggesting that m^6^A modification of these sites is critical for recruitment of SRSF3 and SRSF10 and exclusion of the intron. In contrast, the m^6^A site in Exon2 near the splicing site is critical for removal of SRSF10 and Exon2 inclusion splicing. Therefore, our data highlight a pivotal role of m^6^A modification in RTA pre-mRNA splicing. Interestingly, when the m^6^A sites in both the intron and Exon2 are simultaneously mutated, the level of RTA pre-mRNA drops dramatically (un-published results), suggesting that m^6^A modification also contributes to stability of RTA pre-mRNA. In addition, similar to host mRNAs and HIV-1 transcripts, m^6^A modification may also promote RTA mRNA stability and protein translation through association with m^6^A readers YTHDF1, YTHDF2, and YTHDF3.

Finally, we also found that expression of RTA protein increases the levels of m^6^A modification and promotes its own pre-mRNA splicing. RTA is known to enhance its own transcription^[42]^. Thus, our data for the first time demonstrate that RTA increases its own expression through both transcriptional and post-transcriptional mechanisms. Very interestingly, the KSHV latent protein LANA, which inhibits RTA expression to promote latency^[43]^, suppresses TPA induction of RNA m^6^A modification and inhibits RTA pre-mRNA splicing (un-published results).

In summary, our results not only demonstrate an essential role of m^6^A in regulating RTA pre-mRNA splicing but also suggest that KSHV has evolved two opposite mechanisms to manipulate the host m^6^A machinery to its advantage in promoting lytic replication and latency respectively. This epitranscriptomic mechanism may be used by other herpesviruses as well. Our findings shall shed light on development of new strategies for the control of herpesviral infection.

## Figures and Tables

**Figure 1:**
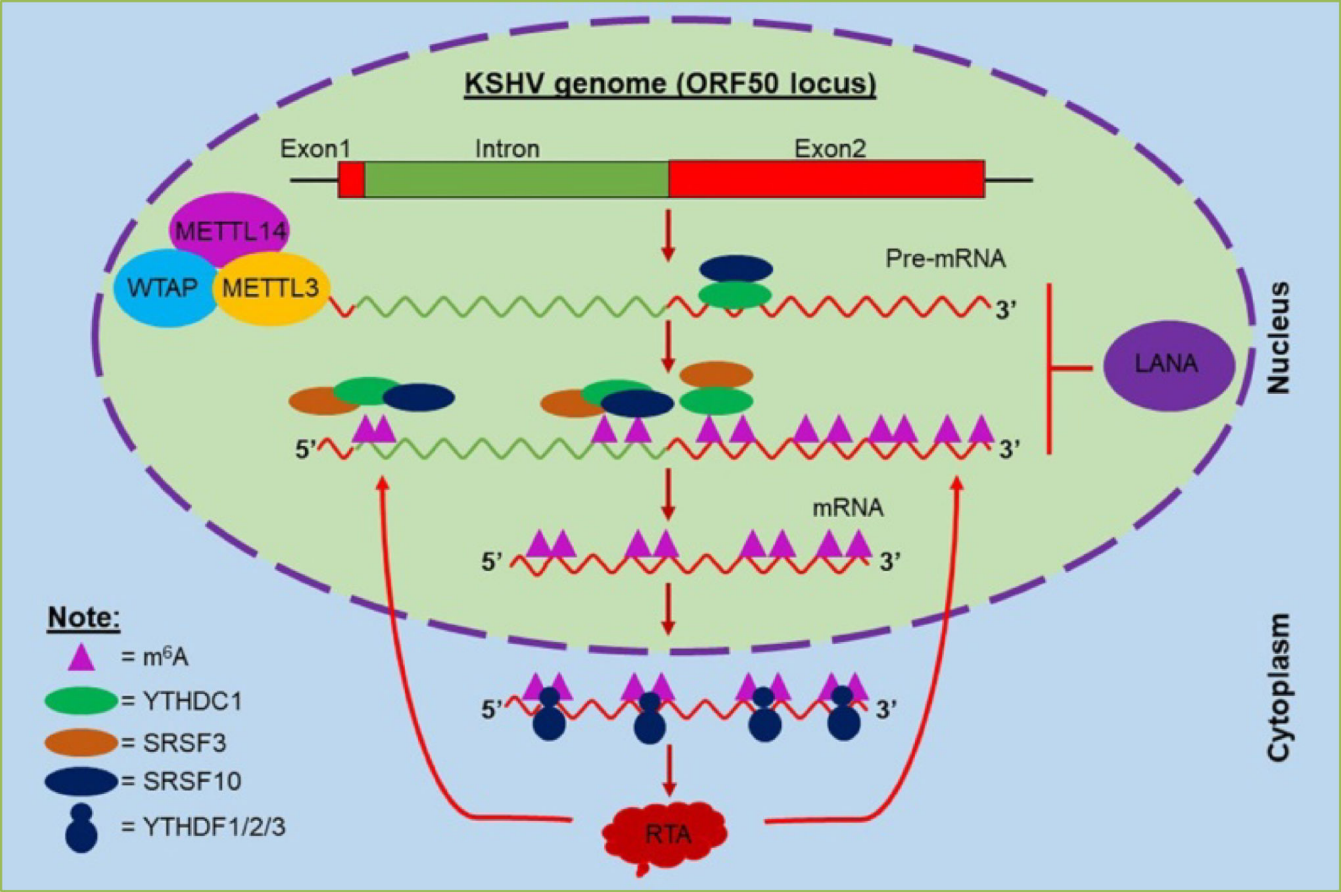
Post-transcriptional m^6^A modification controls KSHV RTA (ORF50) pre-mRNA splicing Multiple m^6^A sites are found in RTA pre-mRNA, which are methylated by m^6^A writers METTL3, METTL14, and WTAP. The m^6^A sites in the intron near the two splicing sites are critical for YTHDC1 binding and recruitment of splicing factors SRSF3 and SRSF10 while the m^6^A site in Exon2 near the splicing site is important for recruitment of SRSF3 and dissociation of SRSF10. Interactions between the m^6^A-modified RTA pre-mRNA and the different splicing factors ensure exclusion splicing of the intron to generate RTA mRNA. The other m^6^A sites may enhance RTA mRNA export, stability, and translation through interaction with m6A readers YTHDF1, YTHDF2, and YTHDF3. The expressed RTA protein enhances the host’s m^6^A machinery to increase the levels of m^6^A to promote its own pre-mRNA splicing and KSHV lytic gene expression. In contrast, KSHV latent protein LANA has the opposite effects on m^6^A and RTA pre-mRNA splicing.

## Abbreviations

KSHV: Kaposi’s sarcoma-associated herpesvirus
KS: Kaposi’s sarcoma
PEL: primary effusion lymphoma
MCD: multicentric Castleman’s disease
m^6^A: N^6^-adenosine methylation
METTL3: methyltransferase like 3
METTL14: methyltransferase like 14
WTAP: Wilms tumor 1 associated protein
FTO: fat mass and obesity associated protein
ALKBH5: AlkB Homolog 5
3’UTR: 3’untranslated region
5’UTR: 5’untranslated region
YTHDC1: YTH domain containing 1 protein
HIV-1: human immunodeficiency virus-1
SRSF3: serine/arginine-rich splicing factor 3
SRSF10: serine/arginine-rich splicing factor 10
RIP: RNA immuno-precipitation
TPA: 12-O-Tetradecanoylphorbol-13-acetate
RTA: regulator of transcription activation
LANA: *latency-associated nuclear antigen*.
